# Temporal evolution and differential patterns of cellular reconstitution after therapy for childhood cancers

**DOI:** 10.1038/s41598-023-31217-3

**Published:** 2023-03-10

**Authors:** Gina Hofmann, Jakob Zierk, Bettina Sobik, Zofia Wotschofsky, Stephanie Sembill, Manuela Krumbholz, Markus Metzler, Axel Karow

**Affiliations:** 1 Department of Pediatrics and Adolescent Medicine, Friedrich-Alexander-Universität Erlangen-Nürnberg (FAU), Erlangen, Germany; 2 Comprehensive Cancer Center Erlangen-EMN (CCC ER-EMN), Erlangen, Germany

**Keywords:** Cancer, Cancer therapy, Chemotherapy, Radiotherapy, Leukopoiesis, Lymphopoiesis, Paediatric research, Medical research, Oncology, Cancer, Paediatric cancer

## Abstract

The cellular reconstitution after childhood cancer therapy is associated with the risk of infection and efficacy of revaccination. Many studies have described the reconstitution after stem cell transplantation (SCT). The recovery after cancer treatment in children who have not undergone SCT has mainly been investigated in acute lymphoblastic leukemia (ALL), less for solid tumors. Here, we have examined the temporal evolution of total leukocyte, neutrophil and lymphocyte counts as surrogate parameters for the post-therapeutic immune recovery in a cohort of n = 52 patients with ALL in comparison to n = 58 patients with Hodgkin’s disease (HD) and n = 22 patients with Ewing sarcoma (ES). Patients with ALL showed an efficient increase in blood counts reaching the age-adjusted lower limits of normal between 4 and 5 months after the end of maintenance therapy. The two groups of patients with HD and ES exhibited a comparably delayed recovery of total leukocytes due to a protracted post-therapeutic lymphopenia which was most pronounced in patients with HD after irradiation. Overall, we observed a clearly more efficient resurgence of total lymphocyte counts in patients aged below 12 years compared to patients aged 12 to 18 years. Our results underline that the kinetics of cellular reconstitution after therapy for HD and ES differ significantly from ALL and depend on treatment regimens and modalities as well as on patient age. This suggests a need for disease, treatment, and age specific recommendations concerning the duration of infection prophylaxis and the timing of revaccination.

## Introduction

Currently, overall cure rates in childhood cancer therapy exceed 80%^1^. Apart from the development of supportive measures and novel targeted therapies, this success is largely still based on the optimized and risk-adapted dosing and scheduling of conventional chemotherapeutic agents. These systemic therapies, however, lead to a suppression of hematopoiesis and general immune functions of varying degrees.

Cellular and immunological reconstitution after cessation of antineoplastic therapy is critical in terms of the risk of infections and the efficacy of revaccinations. In everyday clinical practice, it is generally assumed that patients from about 3 months after termination of antineoplastic therapy can be considered immunocompetent and a low risk of infections as well as an adequate vaccination response can be expected. Accordingly, infection prophylaxis is usually terminated between 3 and 6 months post therapy^2,3^, and the current guidelines recommend the use of inactivated vaccines from 3 months and live vaccines from 6 months after completion of conventional antineoplastic therapy in patients who did not undergo autologous or allogeneic hematopoietic stem cell transplantation (HSCT)^4^. However, these recommendations are rather uniform and not disease-specific, and, in addition to other concomitant factors such as comorbidities, infections, and possible relapse, it can be assumed that the time interval until patients regain immunocompetence also varies between the underlying disease entities and the applied therapeutic regimen.

The highly complex and biologically distinct immunological reconstitution after HSCT has been studied extensively and described in numerous publications for adult and pediatric patients alike^5,6^. Analogous to the prevalence, the cellular and immunologic recovery in pediatric patients who did not undergo allogeneic or autologous stem cell transplantation has been investigated mainly in acute lymphoblastic leukemia (ALL)^7–14^ and less extensively in other disease entities like solid tumors^15–18^.

Therefore, this analysis aimed to illustrate disease- and therapy-dependent differences in the cellular reconstitution after chemotherapy between children and adolescents with ALL and other large and homogenous pediatric patient groups treated for lymphoma and sarcoma, respectively. Consequently, we here retrospectively determined the continuous evolution of total leukocyte, neutrophil and lymphocyte counts as surrogate parameters of immunological recovery from 4 months before until 12 months after the end of conventional chemotherapy in a large pediatric cohort of patients with ALL, patients with Hodgkin`s disease (HD), who had received less intensive lymphotoxic therapy with or without irradiation, and patients who were treated for Ewing sarcoma (ES).

## Results

### Patient characteristics

In total, 132 patients fulfilled the inclusion criteria and could be analyzed in this study. Of these, n = 52 patients were treated for ALL, n = 58 patients were treated for HD and n = 22 patients were treated for ES.

In the group of patients with ALL, 21 individuals (40%) belonged to the standard-risk (SR) group, 26 individuals (50%) belonged to the medium-risk (MR) group and 5 patients (10%) belonged to the high-risk (HR) group. Six patients (12%) experienced a relapse within the study period.

Of the 58 patients with HD, 11 (19%) had been treated according to therapy level (TL) 1, 18 (31%) according to TL-2, and 29 (50%) according to TL-3. Twenty-eight individuals (48%) received additional radiotherapy. The distribution of irradiated patients was even among patients aged ≤ 11 years (47%) and patients aged 12–18 years (54%). Four patients (7%) relapsed within the study period.

Of the 22 patients with ES, 16 individuals (73%) were treated in the R1 arm and 6 (27%) in the R2 arm. Seventeen patients (77%) received additional irradiation of various locations depending on the primary involvement. A relapse within the study period was diagnosed in 4 patients (18%).

The cohort and subcohort characteristics are shown in Table 1.

**Table 1:** Cohort characteristics of n = 52 pediatric patients with ALL, n = 58 pediatric patients with HD, and n = 22 pediatric patients with Ewing sarcoma.

Diagnosis	Number of individuals	Median age at end of chemotherapy [y] (range)	Treatment protocol	Risk/therapy group			Relapse during study period	
Acute lymphoblastic leukemia	52	7 (3–18)	AIEOP-BFM ALL 2009	Non-HR	SR	21	No	46
					MR	26		
				HR		5	Yes	6
Hodgkin's disease	58	14 (3–18)	EuroNet-PHL-C1 EuroNet-PHL-C2 GPOH-HD 2002 Pilot HD-95	TL-1		11	No	54
				TL-2		18		
				TL-3		29	Yes	4
Ewing sarcoma	22	12 (3–18)	EWING 2008 Euro-EWING 99	R1		16	No	18
				R1		6	Yes	4

### Group-specific evolution of total leukocyte, neutrophil, and lymphocyte counts

**Figure 1:**
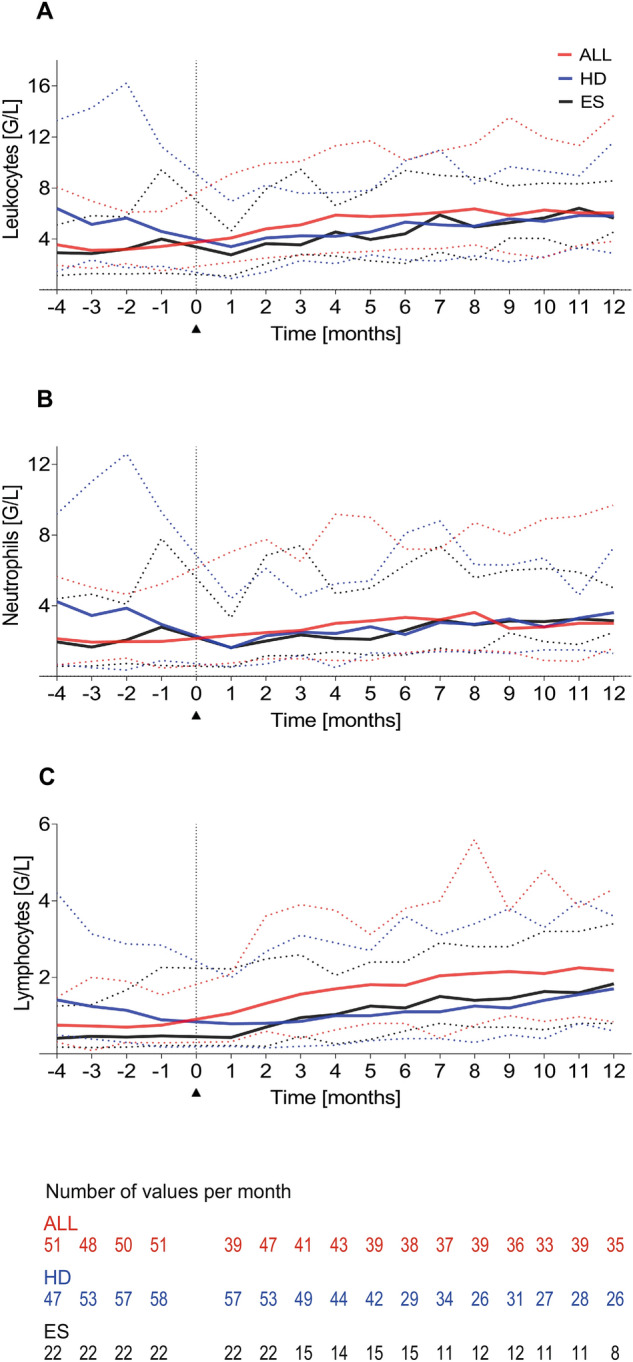
Evolution of the median absolute cell counts from 4 months before until 12 months after the end of chemotherapy for patients with ALL (red), HD (blue), and ES (black). (**A**) total leukocytes, (**B**) total neutrophils, and (**C**) total lymphocytes (1 month corresponding to 4 weeks). Dispersion of values is illustrated by dotted lines.

As depicted in Fig. 1, depending on the diagnostic group, the evolution of total leukocyte, neutrophil, and lymphocyte counts exhibited different characteristics.

Starting from clearly reduced cell counts at the end of maintenance chemotherapy, patients with ALL showed the fastest recovery of all cell types assessed among the three subgroups from month 1 after the end of treatment (Fig. 1A–C). This observation was most evident and consistent for total lymphocyte count (Fig. 1C). No substantial differences in cellular recovery were found when comparing patients with HR-ALL and those with non-HR-ALL (data not shown).

Patients with HD had the highest cell counts 1 month before and 1 month after the end of therapy with median values within the normal age range (Fig. 1A–C). After therapy, total leukocyte, neutrophil, and lymphocyte counts dropped rapidly followed by a protracted post-therapeutic recovery which was most pronounced in lymphocytes as compared to the patients with ALL (Fig. 1C).

The patient group with ES showed the lowest cell counts of the three groups towards the end of chemotherapy. The further course of the total leukocyte and neutrophil count recovery of the subjects with ES after the end of treatment was comparable to the group of patients with HD with a slightly faster increase in the total lymphocyte count (Fig. 1A–C).

### Group-specific cumulative achievement of the age-adjusted lower limit of normal

**Figure 2:**
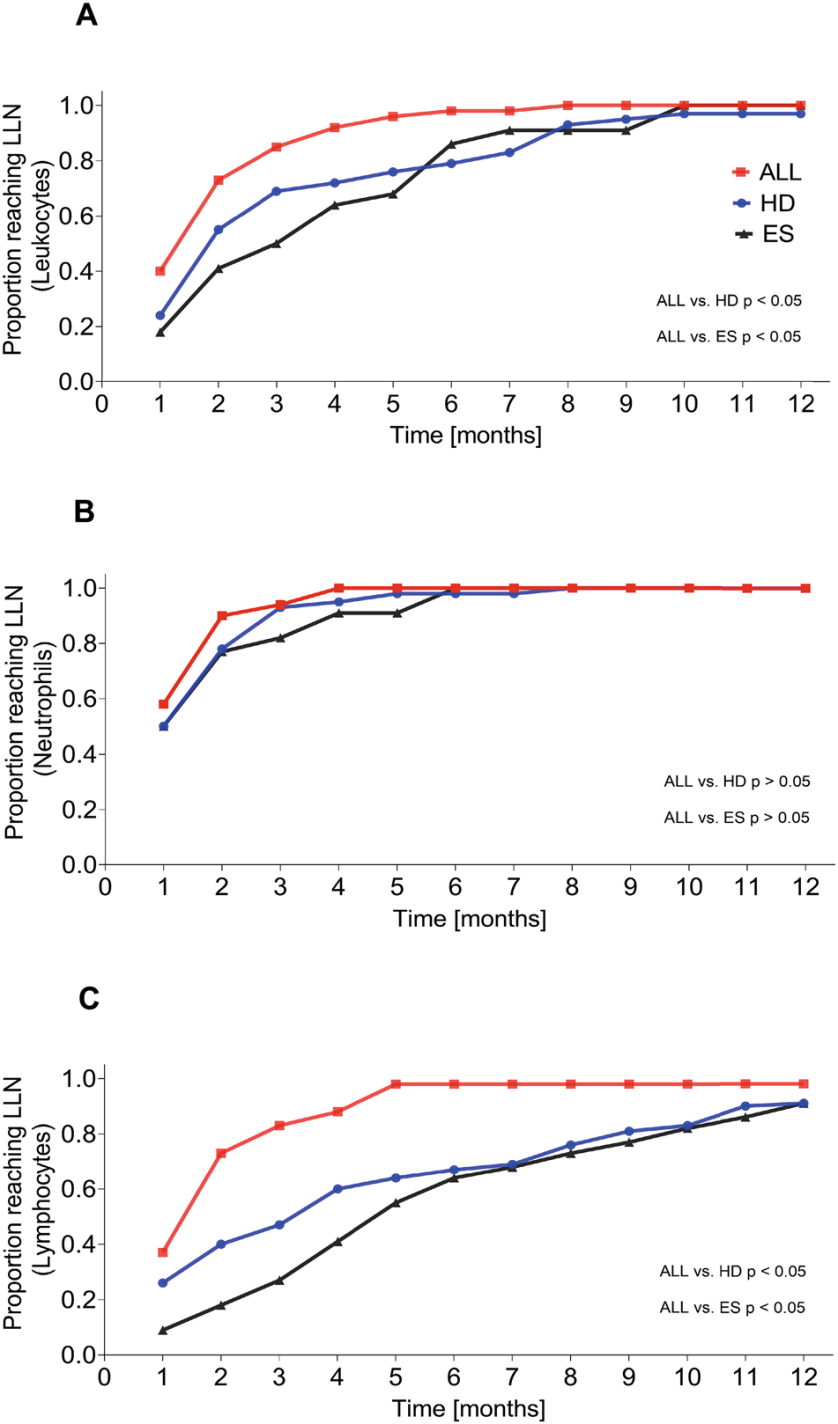
Cumulative achievement of the age-dependent lower limit of normal (LLN) in patients with ALL (red), HD (blue), and ES (black) after the end of chemotherapy for (**A**) total leukocytes, (**B**) total neutrophils, and (**C**) total lymphocytes (1 month corresponding to 4 weeks).

Assessing the cumulative achievement of the age-adjusted LLN after the end of chemotherapy underlined group-dependent differences as illustrated in Fig. 2A–C.

The highest proportion of patients reaching the LLN for total leukocyte count at any time point after the end of chemotherapy until month 12 was observed in the group of individuals with ALL. This distinction reached significance in comparison to patients with HD between 4 and 7 months after chemotherapy (p = 0.002 at month 6) and in comparison to patients with ES between 2 and 5 months after chemotherapy (p = 0.002 at month 5). Children after treatment for ES exhibited the lowest proportion of individuals reaching the LLN for total leukocyte count until month 5 merging with the patients treated for HD thereafter (Fig. 2A).

Whereas no differences were found concerning the proportions of patients reaching the LLN of neutrophil values (Fig. 2B), the most significant disparities between the diagnostic groups affected the cumulative LLN achievement of the total lymphocyte count. Five months after the end of chemotherapy, all patients except for one who had been treated for ALL had reached the total lymphocyte LLN. At this time point, only 63% and 54% of the individuals with HD and ES, respectively, had achieved the LLN for total lymphocyte count (p < 0.001). Notably, even 12 months after the end of chemotherapy, approximately 10% of the patients with HD and ES alike, had not yet reached LLN for lymphocyte count. The intergroup differences and respective significances are illustrated in Fig. 2C.

No age-dependent differences of cellular recovery were found in this cohort (data not shown).

### Subgroup analysis for patients with HD with and without additional radiotherapy

**Figure 3:**
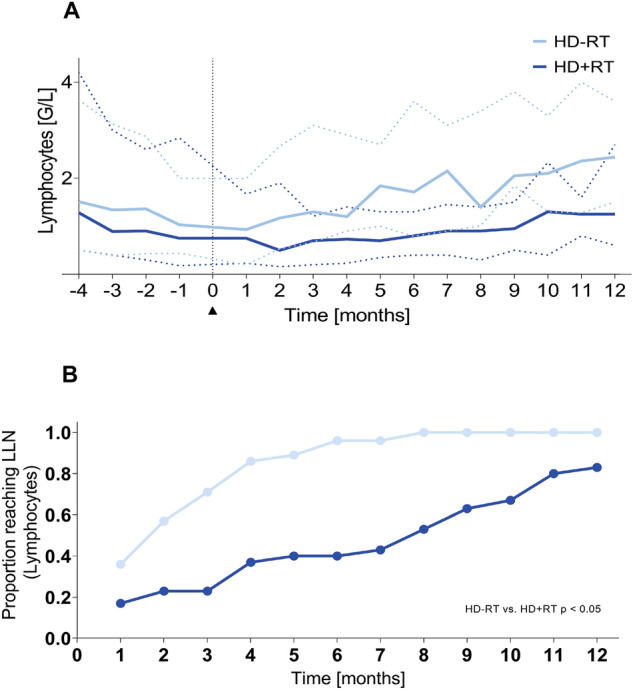
Evolution of the median absolute total lymphocyte counts from 4 months before until 12 months after the end of chemotherapy for irradiated (dark blue; +RT) vs. non-irradiated (light blue; −RT) patients with HD (**A**). Dispersion of values is illustrated by dotted lines. Cumulative achievement of the age-dependent lower limit of normal (LLN) after the end of chemotherapy for total lymphocyte counts in irradiated (continuous dark blue; +RT) vs. non-irradiated (dotted light blue; −RT) patients with HD (**B**).

The separate subcohort analysis comparing patients with HD who had received additional radiotherapy and those who had not received additional radiotherapy showed highly significant differences concerning the recovery of absolute total lymphocyte counts and the consecutive achievement of the total lymphocyte LLN as depicted in Fig. 3A,B. Lymphopenia was significantly more pronounced and long-lasting in individuals who had undergone irradiation compared to those who had not undergone irradiation Fig. 3A). All patients treated for HD without radiotherapy reached the LLN for total lymphocyte count by month 8 after completion of chemotherapy. At the same time point, only 53% of those who had undergone radiotherapy achieved the LLN for total lymphocyte count (p < 0.001). Even after 12 months, only 83% in this latter group accomplished absolute lymphocyte count LLN (Fig. 3B).

Due to the heterogeneity of the localizations of radiotherapy and great disparities in group sizes, no subgroup analysis comparing patients with ES who had received radiotherapy with those who had not received radiotherapy was performed.

### Age-dependent cellular reconstitution

**Figure 4:**
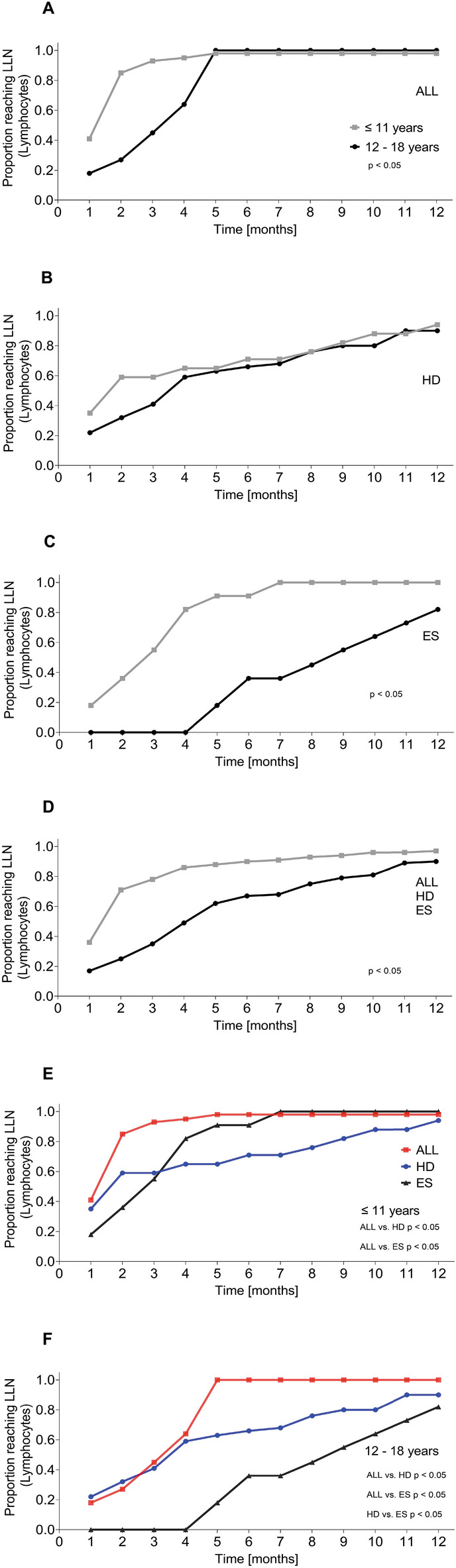
Comparative cumulative achievement of the age-dependent lower limit of normal (LLN) for total lymphocytes after the end of chemotherapy in patients aged ≤ 11 years (grey) and patients aged 12–18 years (black) with ALL (**A**), HD (**B**), ES (**C**), overall including all three disease entities (**D**), in patients aged ≤ 11 years with ALL in comparison with HD and ES (**E**), and in patients aged 12–18 years with ALL in comparison with HD and ES (**F**).

Addressing age dependent patterns of cellular reconstitution, we found that patients aged ≤ 11 years showed a more efficient recovery of the total lymphocyte count regularly reaching the LLN earlier compared to patients aged 12–18 years. Figure 4 illustrates these observations separately for patients with ALL (A), HD (B) and ES (C) and overall for all three disease entities (D). The entity specific differences of total lymphocyte count recovery after therapy described in patients with ALL, HD and ES were still evident in a separate comparison of patients aged ≤ 11 years (Fig. 4E) and patients aged 12–18 years (Fig. 4F).

## Discussion

Childhood cancer and its treatment compromise immune functions bearing implications for the risk of infections and effectiveness of revaccinations. A plethora of studies describe the immune reconstitution after allogeneic HSCT and provide recommendations for infection prophylaxis and revaccination strategies. According to the frequency of the disease, analyses of the immune recovery after the end of therapy in pediatric patients who did not undergo HSCT focused on ALL. In this context, an early report by Alanko et al. described the recovery of blood B-lymphocytes and serum immunoglobulins^7^. The authors found that, based on these parameters, a sufficiently functioning immune system was established 6 months after cessation of chemotherapy and concluded that prophylactic antibiotics can be withdrawn and immunizations started. In a subsequent publication, the same group later described a differential and age-dependent recovery of blood T cell subsets posttherapy^9^. Although their analysis showed a mean reversion to normal values by 6 months, some individual patients continued to have subnormal values for up to 1 year after therapy, some of whom exhibited increased susceptibility to infections. Such observations were largely confirmed in later studies in ALL^11,14,19,20^. Some of these analyses showed that in comparison, B-cells were reduced more significantly and for longer periods than T-cells. In a long-term follow-up, van Tilburg and colleagues showed that whereas naive B and T cells exhibited a relatively fast recovery, memory B-cells regenerated significantly slower and memory T-cells did not fully recover during the entire 5-year follow-up^12^. In a more recent large study including 116 participants, Williams and colleagues underlined that immune reconstitution differs between lymphocyte compartments^21^. Concerning the influence of treatment intensity, Ek et al. found that immune reconstitution after childhood ALL was most severely affected in the high-risk group^10^.

The immune reconstitution after therapy for solid tumors in children and adolescents has been characterized less extensively as compared to ALL.

In an early orienting pilot study, Cranendonk et al. retrospectively determined the effects of various chemotherapeutic drug regimens on the numbers of different blood cell types in 131 children treated for solid tumors. Apart from the general cytoreductive effects during treatment, they observed that, in the majority of the children, the lymphocyte count became normal between 1 and 12 months after cessation of therapy^22^. Later, Alanko and coworkers focused on the hematologic and immunologic recovery in a small analysis including 11 children who had been treated for HD (n = 3), Nephroblastoma (n = 4), Burkitt`s Lymphoma (n = 2), clear cell sarcoma (n = 1) and rhabdomyosarcoma (n = 1). The group described that the lymphocyte counts of most patients normalized during the first 12 months after therapy. The recovery in patients with HD or Burkitt`s lymphoma was slower than in patients with nephroblastoma and radiotherapy appeared to prolong immune reconstitution^15^. In a later investigation, Kovacs and colleagues assessed immune recovery in 88 children receiving chemotherapy for ALL (n = 43), lymphoma (n = 15), bone tumors (n = 20), and other solid tumors (n = 10). The group determined serum immunoglobulin levels (Ig), natural killer activity (NK), antibody-dependent cellular cytotoxicity (ADCC), and T and B cell proliferation 1 year after cessation of therapy. They found, that cytotoxic therapy can lead to long-term depression of the immune system, which was most prominent in patients with ALL.

For adults with HD, a treatment-associated marked long-term dysregulation of T-cell subset homeostasis has long been described^17^ and it had been shown that radiation therapy decreases the absolute CD4 T-cell counts^23^. As a consequence, antibody response to pneumococcal vaccine was profoundly impaired in patients who had received intensive treatment for HD^24^.

Comparable larger studies in pediatric cohorts have not been performed.

To illustrate varying characteristics of the cellular reconstitution after completion of chemotherapy in pediatric patients with ALL and other disease entities, we here present a comparative analysis of the course of total leukocyte, neutrophil and lymphocyte counts from 4 months before until twelve months after therapy in a large pediatric cohort of n = 132 patients who had been treated for ALL, HD and ES. Depending on the underlying disease and the resulting treatment regimens and modalities, we describe significant differences mainly affecting the total lymphocyte counts.

In contrast to earlier reports^10^, we found no differences in the cellular reconstitution between patients with ALL belonging to the HR group and those belonging to the MR or SR group. This might be explained, however, by the relatively small proportion of patients with HR in this study.

In particular, we saw a marked and prolonged post-therapeutic lymphocyte depression in patients with HD and ES as compared to patients with ALL. Lymphopenia was most distinct in individuals with HD who had received radiation therapy. This appears obvious because in the vast majority of cases the radiation field comprised the mediastinum and thus the thymic region with direct influence on T lymphocyte regeneration. In patients with HD, these findings are in line with earlier investigations in adult cohorts^17,23,24^.

In addition to these entity, treatment, and modality specific differences in cellular reconstitution, we observed a clear age dependency with patients aged ≤ 11 years showing an earlier recovery of the total lymphocyte count than patients aged 12–18 years. An age dependency of the post-therapeutic immune reconstitution has been proposed in different contexts in some earlier studies^9,25^ whereas others did not report comparable differences^12^. Considering the lower median age of patients with ALL compared to patients with HD and ES, this finding could contribute to the differences in lymphocyte reconstitution described between the disease entities in this study.

Overall, our study underlines that immune reconstitution after chemotherapy for childhood cancer highly depends on the underlying disease entity, the therapeutic regimen, and treatment modality as well as patient age and differs significantly between ALL and solid tumors. These discrepancies might at least in part be a consequence of the less intensive maintenance therapy applied in ALL but not in HD or ES. However, at the same time, current recommendations for infection prophylaxis and revaccination in childhood cancer have been mainly derived from pediatric patients with ALL^4,26–28^, are still rather uniform, and do not consider potential differences for patients with solid tumors. In pediatric patients, who have been treated for childhood cancers without autologous or allogeneic HSCT, infection prophylaxis is generally withdrawn between 3- and 6 months^2,3^, and inactivated vaccines are administered from 3 months and live vaccines from 6 months after cessation of conventional antineoplastic therapy^4^.

The results of our study could potentially contribute to discussions about adjusting the recommendations and establishing differentiated disease-, therapy- and modality-specific guidelines considering the differences in cellular reconstitution and in particular the clear and long-lasting effect of irradiation on lymphocyte total counts.

We are aware that our study bears some limitations. Above all, due to the retrospective design, we could not investigate any additional immunological parameters and therefore focused on absolute leukocyte, neutrophil, and lymphocyte blood counts as surrogate parameters of immunological recovery. Moreover, we could not draw any clinical correlations, especially between the evolution of cell counts and infectious complications. In addition, the recent implementation of the bispecific T cell engager blinatumomab in the treatment protocols might further influence and thus alter cellular reconstitution after therapy in patients with ALL.

As a consequence, future joint large studies are essential to cover these aspects in a disease- and treatment-specific manner. For a few treatment protocols, such investigations have already been initiated.

## Patients and methods

### Study design

This retrospective study was conducted in a single pediatric cancer center in accordance with the declaration of Helsinki. It used routine clinical data acquired during patient care, which was pseudonymized for the collection and subsequently anonymized for the analyses.

As also confirmed by the institutional ethic committee of the Friedrich-Alexander-Universität Erlangen-Nürnberg, analysis of test results performed during patient care for research, like in this study, is in accordance with the applicable German/Bavarian regulations and does not require patients’ explicit consent nor a separate ethical approval.

#### .

### Patients

Patients aged 0–17 years at the time of diagnosis who were treated at our center between the years 2000 and 2019 belonging to one of the following groups were eligible for this study:

- Individuals with ALL treated according to the protocol AIEOP-BFM ALL 2009
- Individuals with HD treated according to the protocols EuroNet-PHL-C2, EuroNet-PHL-C1, GPOH-HD 2002, and HD 95
- Individuals with ES treated according to the protocols EWING 2008 and EWING 99.

According to the treatment protocol AIEOP-BFM ALL 2009, stratification into the high risk group was based on prednisone poor-response on day 8, ≥ 10% blasts by flowcytometry on day 15, non-remission on day 33, positivity for MLL/AF4 gene rearrangement or translocation t(4;11), hypodiploidy (< 45 chromosomes) or inadequate MRD PCR response^29^. In the protocols EuroNet-PHL-C2, EuroNet-PHL-C1, GPOH-HD 2002, and HD 95, therapy levels TL-1, TL-2 and TL-3 were assigned according to the disease stage and indication for additional radiotherapy was based on early response after two initial cycles of chemotherapy^30–32^. Risk groups R1, R2, and R3 according to the protocols EWING 2008 and EWING 99 were based on initial tumor stage, tumor volume and histologic response to neoadjuvant chemotherapy^33^.

Patients who underwent autologous or allogeneic hematopoietic stem cell transplantation as first-line therapy were excluded from the analyses. Patients experiencing a relapse within the study period were eligible until the date of relapse diagnosis. Blood counts after this date were not considered. Individuals who deceased during the study period were also excluded.

### Methods

The data sets were extracted from patients’ digital and paper records and the laboratory information system. Patient groups were categorized according to the underlying disease with respect to risk group, treatment protocol, therapy level, and treatment modalities including chemo- and radiotherapy. The study period was defined as 4 months before and 12 months after the end of chemotherapy, 1 month corresponding to 4 weeks. All machine and manual blood counts performed in our hospital during the study period including total leukocytes, neutrophils, lymphocytes, segmented neutrophils, and band neutrophils were exported from the central laboratory information system swisslab. Where available, manually determined blood cell counts were used for this analysis. Total neutrophil counts comprised the sum of segmented and band neutrophils. In case, no manually counted blood cell values were available, device counted values were employed. If more than one manually or device counted blood cell value was determined from a patient on the same day, the daily mean value was calculated and included. A monthly mean of leukocyte, neutrophil, and lymphocyte count was determined for each patient. These values were then merged according to the diagnostic categories and subcohorts resulting in a subgroup-specific monthly median with range. Age-dependent reference intervals including the lower limit of normal (LLN) for total leukocyte counts were adopted from Zierk et al.^34^. Other reference intervals were used according to Soldin et al.^35^. Subgroup division for age dependent analyses was also based on data reported in these latter studies. For cumulative analyses, the proportion of patients who had reached LLN before or at the specific time points was calculated resulting in values from 0 (0%) to 1 (100%).

### Statistical analyses

The groups were tested against each other using Fisher ‘s exact test. Results with a two-tailed p-value < 0.05 were considered significant. The following groups were tested against one another in each blood cell category: HR-ALL (n = 5) vs. non-HR-ALL (n = 47) (1), HD-TL1 (n = 11) vs. HD-TL2 (n = 18) (2), HD-TL1 (n = 11) vs. HD-TL3 (n = 29) (3), HD-TL2 (n = 18) vs. HD-TL3 (n = 29) (4), HD non-irradiated (n = 28) vs. HD irradiated (n = 30) (5), ES-R1 (n = 16) vs. ES-R2 (n = 6) (6), ALL total (n = 52) vs. HD total (n = 58) (7), ALL total (n = 52) vs. ES total (n = 22) (8), HD total (n = 58) vs. ES total (n = 22) (9), ALL ≤ 11 years (n = 41) vs. ALL 12–18 years (n = 11), HD ≤ 11 years (n = 17) vs. HD 12–18 years (n = 41), ES ≤ 11 years (n = 11) vs. ES 12–18 years (n = 11), total patients ≤ 11 years (n = 69) vs. total patients 12–18 years (n = 63), ALL ≤ 11 years (n = 41) vs HD ≤ 11 years (n = 17) vs. ES ≤ 11 years (n = 11), ALL 12–18 years (n = 11) vs. HD 12–18 years (n = 41) vs. ES 12–18 years (n = 11).

The cumulative incidence of reaching LLN for the specific time points was calculated as the number of patients who had reached LLN divided by the total number of individuals in the group.

## Data Availability

The data presented in this study are available from the corresponding author on reasonable request. The data are not publicly available due to privacy and ethical restrictions.
